# Successful multimodality treatment of anorectal melanoma after wide local excision: a case report

**DOI:** 10.3389/fonc.2025.1535376

**Published:** 2025-06-26

**Authors:** Zhao Liang, Liu Zongjian, Pan Chunlai, Wang Lihua, Yuan Hexue

**Affiliations:** ^1^ Department of Colorectal Anal Surgery, Shenyang Coloproctology Hospital, Shenyang, Liaoning, China; ^2^ Department of Pathology, Shenyang Coloproctology Hospital, Shenyang, Liaoning, China

**Keywords:** anorectal tumor, melanoma, targeted therapy, immunotherapy, case report

## Abstract

**Objective:**

The purpose of this case report is to explore the efficacy of comprehensive treatment of anal melanoma and to provide reference for the treatment of anal melanoma.

**Methods:**

A rare case of anorectal melanoma was collected. After local excision, the patient was treated with a combination of the anti-vascular survival drug bevacizumab, anti-PD-1 immunotherapy and other treatments, and the patient’s prognosis and survival were observed.

**Results:**

A 61-year-old male was admitted to the hospital with complaints of anal prolapse and a mass appearing after defecation, accompanied by intermittent bloody stool for one week. He was initially diagnosed with mixed hemorrhoids, and a hemorrhoidectomy was performed. Postoperative pathology confirmed malignant melanoma with negative surgical margins, leading to a clinical diagnosis of AM. Following local excision surgery, the patient underwent targeted therapy with bevacizumab, anti-PD-1 immunotherapy and other treatments. After nearly three years of follow-up, the patient remained in good condition and while follow-up CT and MRI revealed some enlarged lymph nodes, there were no symptoms or signs of systemic metastasis.

**Conclusion:**

The combination of local resection of the primary lesion with bevacizumab targeted therapy and anti-PD-1 immunotherapy is feasible and can substantially improve the survival time of patients with AM.

## Introduction

Anorectal melanoma (AM) is a rare tumor with an incidence rate of 4.8 per 10 million annually (1). It often presents with non-specific symptoms, such as an anal mass, pain, or blood in the stool. Diagnosing AM can be challenging, as clinical and imaging findings are typically inconclusive, leading to frequent misdiagnoses and delays in early detection. Studies have shown that approximately 60% of patients with AM present with distant metastases at the time of diagnosis (2). Further research has reported a 5-year survival rate of 6-22% (3), with advanced-stage disease having a 5-year survival rate of 0% (4). Herein, we report a rare case of AM, analyzing the pathological and molecular characteristics, as well as the treatment approach, to enhance understanding of the disease, provide a reference for clinical multidisciplinary treatment, and promote further targeted research.

## Case report

A 61-year-old Chinese man presented to our hospital with a one-week history of intermittent bloody stools. The patient was admitted to the hospital with a one-week history of intermittent hematochezia, accompanied by persistent swelling and pain in the anus. There was no fever, weight loss, or fatigue. The patient reported normal diet and sleep patterns, with regular urination and stools (1–2 times per day), although the stools were not well formed. The patient had a history of hypertension for 10 years. The patient had a 40-year history of smoking one pack per day and consuming half-pound wine daily. He denied any family history of the disease. Physical examination revealed that the patient had a normal body mass index (BMI). No palpable or enlarged superficial lymph nodes were noted. Cardiopulmonary function was normal, and the liver and spleen were not enlarged on palpation. Specialized examination showed irregular perianal skin tags, with the largest measuring approximately 0.5 × 0.6 cm and maximum thickness 0.4 cm. Lung CT and ultrasound examinations of the liver, gallbladder, and spleen showed no significant abnormalities. Anorectal endosonography also revealed no obvious abnormalities.

The patient was misdiagnosed with mixed hemorrhoids and underwent a mixed hemorrhoidectomy on August 11, 2021 to remove an external hemorrhoidal mass measuring approximately 0.5x0.6 cm and maximum thickness 0.4 cm. However, postoperative pathology findings: AM, mitotic index was 1/mm^2^, and no lymph node or vascular invasion was observed.

**Figure 1:**
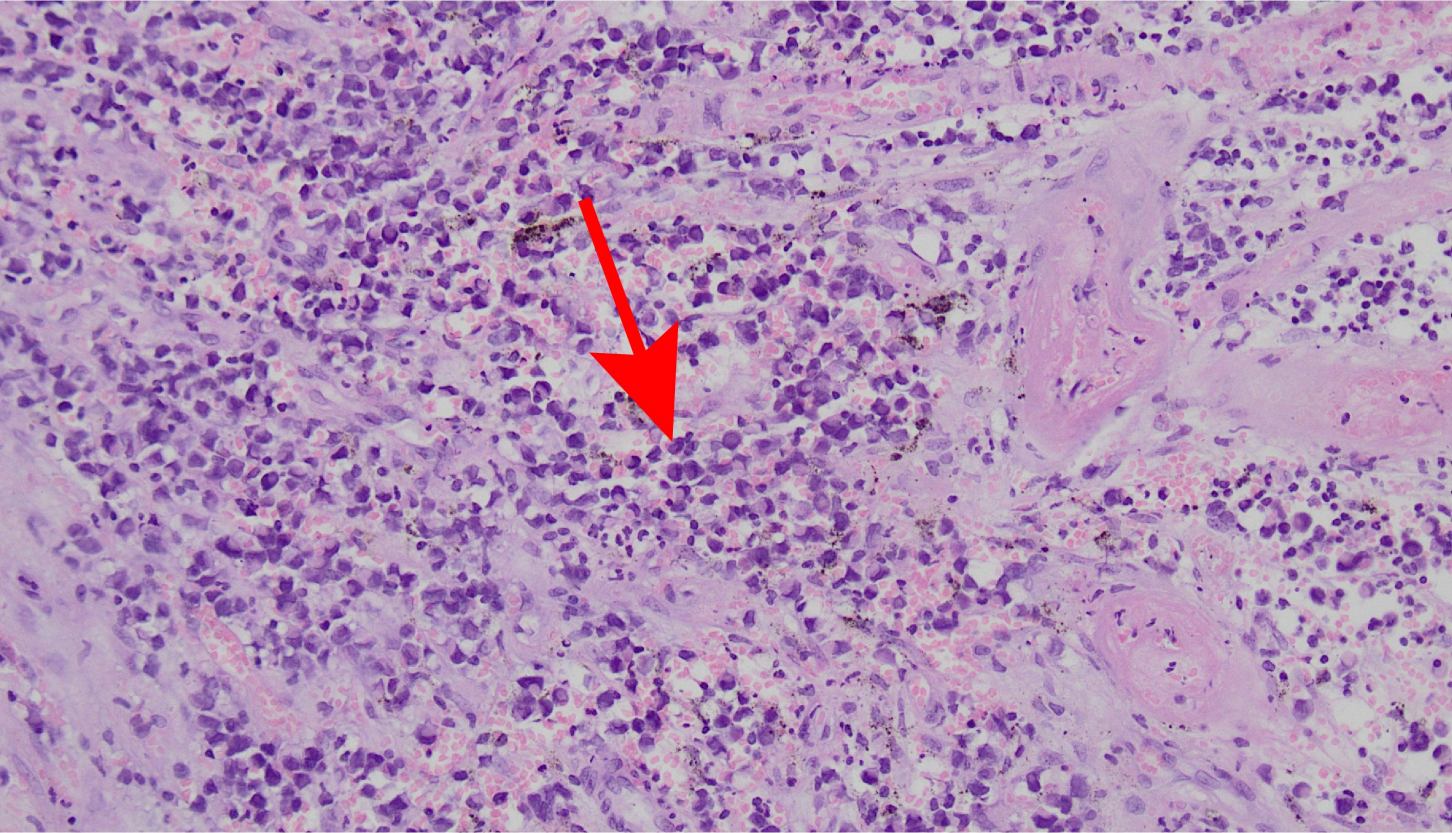
Hematoxylin and eosin-stained images show marked melanin, infiltration of surrounding tissue, heterogeneous tumor cells, abundant mitosis with atypical mitosis, and acidophilic, basophilic, rhabdomyoloid cytoplasm (×200).

**Figure 2:**
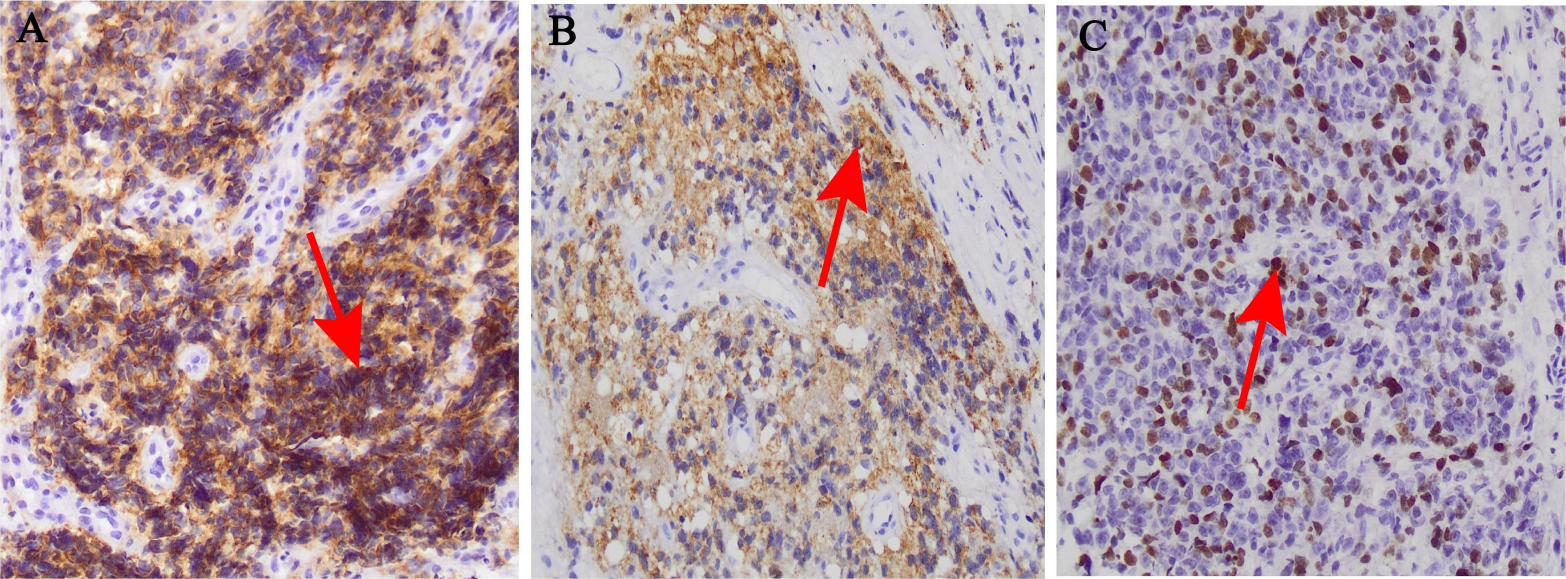
Histological and immunohistochemical images (×200). **(A)** CD117 diffuse positive reaction; **(B)** HBM45positive reaction; **(C)** Ki67 positive reaction.

**Figure 3:**
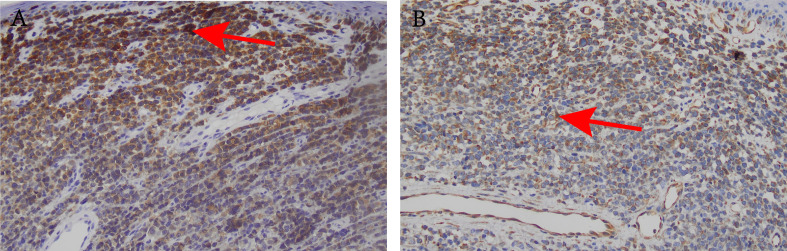
Histological and immunohistochemical images (×200). **(A)** Mel-A positive reaction; **(B)** Vim positive reaction.

Pathological analysis revealed a negative resection margin. Postoperative laboratory tests showed that tumor markers CA-50, CA24-2, carcinoembryonic antigen and alpha-fetoprotein were normal, while CA72–4 was elevated. Hematoxylin and eosin staining of the pathological tissue confirmed the diagnosis of anorectal melanoma (**Figure 1**). Immunohistochemical staining revealed positive results for Vim, HMB45, Mel-A, CD117, CD56, CD34 (blood vessels), and Ki67 (50%) (**Figures 2**, **3**). Therefore, the final diagnosis was anal melanoma (AM). After a multidisciplinary consultation, the patient started a 4-course chemotherapy on September 3, 2021, with intravenous cisplatin (30 mg per m^2^) and oral temozolomide (150 mg per m^2^) and once in 21 days. Three months later, abdominal enhanced computed tomography (CT) and rectal magnetic resonance imaging (MRI) revealed enlarged lymph nodes in the right and left internal iliac regions, with the largest measuring approximately 17 × 21 mm which lymph node metastasis was considered. Genetic testing did not detect BRAF V600E mutation, and the patient subsequently received five courses of intravenous bevacizumab (15 mg per square meter) plus nivolumab (3 mg per kilogram) every 21 days. At the last follow-up, in July 2024, the patient was in good physical condition, with no abnormalities on physical examination. From February 28, 2022 to July 3, 2023, the patient underwent CT and MRI every 1 ~ 2 months, which showed no significant changes in the enlarged pelvic lymph nodes and no evidence of metastasis to other sites. The patient survived well and no adverse events or treatment-related toxicities were noted.

## Discussion

AM is a rare tumor, with the majority of malignant melanocytes originating in the anal canal, and a smaller proportion originating in the lower rectum and rectosigmoid junction (5). It is reported that the overall median time to diagnosis was 4 months for anal melanoma, 3 months for rectal melanoma, and 4 months for anorectal junction melanoma (6). Thus, the disease is characterized by early systemic spread and generally has a poor prognosis.

AM often presents as polypoid lesions and is easily misdiagnosed as hemorrhoids or polyps. Some studies have found that distant metastasis occurred in 6 out of 13 (46.2%) patients with AM who were initially misdiagnosed as hemorrhoids. Further research has shown that such misdiagnosis can significantly impact the prognosis of the disease (5). In the present case, after being misdiagnosed as mixed hemorrhoids, local excision (LE) of the lesion was performed. The patient was treated with cisplatin combined with oral temozolomide chemotherapy, and the pelvic lymph nodes were enlarged significantly. In order to explore the further therapeutic options, genetic testing was performed and no BRAF V600E mutation was found, so bevacizumab targeted therapy combined with anti-pd-1 immunotherapy was used. The patient was followed up regularly for 3 years and remained in good general health. Due to the rarity and aggressiveness of AM, there are no established standard diagnostic and therapeutic approaches to date. Therefore, this case could provide valuable guidance and reference for the management of patients with AM.

Currently, evidence suggests that tumor stage in melanoma may be an independent predictor of survival (7, 8). Nonetheless, a meta-analysis of 347 cases found that survival in AM was not associated with tumor staging (1). Although a separate AJCC staging system exists for head and neck mucosal melanoma (9), there are no specific staging criteria for AM. In addition, some studies have indicated that patients with AM lesions ≤2 mm have better survival outcomes than those with lesions >2 mm (10, 11). Surprisingly, several reports have found that tumor size in AM are not associated with disease-specific survival (12, 13). These findings suggest that there is still insufficient evidence to propose definitive treatment guidelines for AM. Therefore, treatment options and therapeutic approaches should be carefully selected on a case-by-case basis.

So far, multimodal treatment involving surgical therapy has become the primary treatment strategy for AM. Surgical options for AM can be classified into abdominal perineal resection (APR) and local excision (LE) for anus preservation. There is ongoing controversy between the two approaches, but both aim to improve survival through R0 resection. APR is a more invasive procedure, with the disadvantages of a longer hospital stay and extended recovery period (14–16). Furthermore, the burden of colostomy often negatively impacts the quality of life (14–16). APR is also associated with a higher rate of complications, such as voiding problems and sexual dysfunction (14–16). In contrast, LE is a less invasive procedure that is becoming increasingly popular. Notably, several studies have confirmed that APR has the same 5-year survival and recurrence rates as LE (17), suggesting that LE should be considered an initial treatment option for AM (4, 12, 18). In the present case, it was demonstrated that the surgical approach of LE can achieve comparable survival and local control results.

Historically, patients with AM have responded poorly to radiotherapy and/or chemotherapy (19). To date, no systemic therapeutic regimen has been established as the standard of care for AM. However, with the development of targeted therapies (such as BRAF, MEK, CDK4/6, and C-KIT inhibitors) and immunotherapies (including anti-CTLA4 antibodies and anti-PD-1 antibodies), the systemic treatment of melanoma has been dramatically revolutionized (20).

It has been reported that activating mutations in BRAF or c-KIT may be present in malignant melanoma, which has important implications for the tumor’s response to anticancer drugs targeting BRAF or c-KIT (21). Further studies have shown that in patients with BRAF-mutated metastatic melanoma, the combination of the BRAF inhibitor Dabrafenib and the MEK inhibitor Trametinib may be an effective therapeutic modality, demonstrating impressive remission rates and survival benefits (22). However, in this case report, since BRAF V600E was not mutated, the patient was treated with the angiogenesis-inhibiting drug Bevacizumab. Bevacizumab is a humanized monoclonal antibody and was the first anti-angiogenic drug used in antitumor therapy, showing promising efficacy in the treatment of anorectal melanoma (23). Furthermore, researchers have conducted several *in vivo* studies and found that Bevacizumab not only inhibits tumor angiogenesis but also enhances the efficacy of cytotoxic drugs and immunotherapies, thereby exerting significant antitumor effects (24).

With the discovery of immune checkpoints, cancer immunotherapy has emerged as an effective treatment modality for various solid malignancies (25). Monoclonal antibodies that block immune checkpoint receptors, such as cytotoxic T-lymphocyte antigen (CTLA)-4, programmed death (PD)-1, and its ligand PD-L1, have shown broad-spectrum activity against a wide range of tumor types, leading to prolonged survival in many patients. Numerous studies have demonstrated the clinical success of immune checkpoint inhibitors (ICIs) (26–28). The first immune checkpoint inhibitor to receive approval in 2011 was the anti-CTLA-4 antibody ipilimumab for the treatment of unresectable or metastatic melanoma (29). Subsequently, the anti-PD-1 drugs nivolumab and pembrolizumab were approved for the treatment of melanoma (29). In the present case, the patient received immunotherapy with nivolumab and achieved good clinical remission. Despite the significant potential of immune checkpoint inhibitors, their success is somewhat limited by the occurrence of inflammatory toxicities, collectively known as immune-related adverse events (30), which can lead to treatment delays and interruptions. Nevertheless, combining immunotherapy with chemotherapy or targeted therapy has become a common approach in the treatment of AM (23).

Notably, angiogenic factors contribute to immunosuppression by directly inhibiting antigen-presenting cells and immune effector cells or by enhancing the activity of regulatory T cells (Tregs), myeloid-derived suppressor cells (MDSCs), and tumor-associated macrophages (TAMs). These suppressive immune cells, in turn, can promote angiogenesis, creating a vicious cycle of impaired immune activation (24). Surprisingly, it has been demonstrated that in advanced melanoma, the combination of bevacizumab and ipilimumab has shown favorable effects on anti-angiogenesis and ICIs, resulting in prolonged patient survival (31). However, nivolumab adjuvant therapy in AM patients has been associated with significantly longer recurrence-free survival and a lower incidence of grade 3 or 4 adverse events compared with ipilimumab (28). Herein, after LE of the tumor, the patient remained alive and in good condition following the combination of adjuvant therapy with bevacizumab and nivolumab, with a follow-up period of 3 years. The results of this case report further confirm that the combination of the antiangiogenic agent bevacizumab and the anti-PD-1 agent nivolumab can prolong survival in AM patients and provide a reference for developing a standard treatment regimen for AM.

## Conclusion

This article reports a rare case of AM and its surgical treatment modality, followed by postoperative adjuvant therapy with the combination of the antiangiogenic drug bevacizumab and the anti-PD-1 drug nivolumab, a strategy that has been seldom reported to date. Moreover, we reviewed the relevant literature and discussed the choice of surgical treatment for AM. Notably, evidence in the literature suggests that LE can achieve comparable survival and local control outcomes when compared with APR. In addition, there is growing support for the strategy of combining bevacizumab and ICIs with favorable clinical activity. Collectively, we found that the combination of bevacizumab and nivolumab significantly improved the survival of our patient. However, few case reports regarding this treatment strategy and large-sample, multicenter studies are severely lacking. Thus, further case studies with long-term follow-up are needed to confirm these results.

## Data Availability

The raw data supporting the conclusions of this article will be made available by the authors, without undue reservation.
